# Rabies control in Nepal: a missed opportunity

**DOI:** 10.3389/fvets.2023.1184371

**Published:** 2023-09-20

**Authors:** Krishna Prasad Acharya, Rosie Kwon, Seong Ho Cho, Dong Keon Yon

**Affiliations:** ^1^Animal Quarantine Office Kathmandu, Department of Livestock Services (DLS), Kathmandu, Nepal; ^2^Department of Biomedical Engineering, University of Michigan, Ann Arbor, MI, United States; ^3^Division of Allergy-Immunology, University of South Florida Morsani College of Medicine, Tampa, FL, United States; ^4^Kyung Hee University Medical Center, Center for Digital Health, Medical Science Research Institute, Kyung Hee University College of Medicine, Seoul, Republic of Korea

**Keywords:** rabies, one health approach, public health, challenges, opportunities, Nepal

Nepal is committed to end human deaths from dog-mediated rabies by 2030. Nepal has recognized success in reducing the number of human deaths from rabies, with the human fatalities decreasing from 97 in 2008/09 to 32 in 2017/18 (1). However, the incidence of rabies is supposed to be higher than reported due to widespread underreporting of cases of rabies (2), attributed to a lack of awareness among the general public regarding the mandatory reporting of cases of rabies. The incidence of rabies has not, however, been reduced as forethoughted in policies and plans related to rabies control. This failure has been due to multifactorial issues, which have often been neglected in current rabies control programs. A few concerns related to disease control, such as lack of cross-sectoral collaboration, have been previously discussed (1–3); however, a few issues such as the role of ecological and socioeconomic determinants have not been discussed yet. Thus, in this commentary, the missing approaches regarding rabies control in Nepal will be further discussed.

The Nepalese government lacks a scientific, realistic and systematic rabies control policy and strategy (2), which has stymied the rabies control programs in the country. Despite the lack of a national rabies control policy, some preventive and control measures focusing on awareness, mass vaccination of free-roaming and domestic dogs and cats, and animal birth control are in place (1, 2, 4). These programs have been concentrated mainly in urban areas, and the rural areas with the highest rabies burden are out of reach of such control measures (2). Furthermore, these preventive and control measures have missed the opportunity to take into account the role of wild (sylvatic) animals, such as foxes and wolves, in rabies virus transmission. The local socioeconomic factors such as low literacy rate, low income, and close contact with feral dogs and poor hygiene and sanitation that increase the risk of acquiring rabies virus and further transmission in Nepal have also been largely overlooked. The local ecological conditions such as the locality's climate and proximity to wildlife not only influence the incidence and burden of rabies in any particular locality but could also act as drivers in rabies virus transmission. Consequently, attention to these factors could have helped devise more effective rabies control programs in Nepal.

Considering the diverse eco-climatic conditions and lack of research, the epidemiology of rabies in the Nepalese context is still not well-understood. There are a few funded studies on rabies, but these studies are passive epidemiological reporting of cases of rabies and do not add substantial information to inform the national rabies control program in Nepal. So far, only four cases of rabies in Jackals, a wild animal, have been reported (1)—with the previous two in years-2016 and 2017 and the recent two in 2023 (Unpublished). This low number of records of cases of rabies could be due to either underreporting or the fact that the actual burden of rabies in wild animals is lower than in domestic animals. Thus, it is necessary to have in-depth research to look at the data concerning how many animals and human deaths are from rabies and how many of these are due to transmission from wildlife. It is risky to shift the focus away from canine immunization and rabies control in the absence of strong evidence to support wildlife as a driver of rabies transmission. The national rabies control programs should include research on the significance of ecological factors on the incidence of rabies. This would have been used to develop-region-specific rabies control programs. The current national rabies control programs, without considering ecological views, might seem effective in the short run; however, in the long-term, they will not be sustainable, principally due to changes in pathogen dynamics associated with climate change.

From the social perspective, social characteristics such as economic status, literacy rate, close contact with feral dogs and poor sanitation and hygiene affect rabies virus transmission pathways. Control programs that fail to consider the prevailing social conditions in a particular location may fail to fetch the intended result. Even today, many people in the rural areas of Nepal resort to traditional healers and samans rather than visiting healthcare facilities to cure. People usually apply herbs on bite wounds rather than cleaning and disinfecting bitten wounds. For instance, juice prepared from grinding leaves of *Aconitum chaomanthum*, and *Aconitum ferox* were reported to have been applied in the bite wound to prevent rabies after a dog bite and the flowers and stems of *Cissens repens* were ground together and one teaspoonful of powder obtained was drunk with water with a false optimism as a cure for rabies (5). Similarly, in another study in Far-Western Nepal, local people allegedly used a leaf paste of *Bauhinia purpurea L., Fabaceae* to the dog bite wound to prevent rabies occurrence [Devkota and Karmacharya as cited in (6)]. The efficacy of these traditional ethnomedicinal practices has not been scientifically investigated and is highly likely to have little or no effect. This practice of applying herbs to bite wounds rather than cleaning and disinfecting and following post-exposure prophylaxis might result in the complication of bite wounds and deaths due to rabies.

The local ecological factors may have been crucial in the transmission of rabies in Nepal as evinced by the majority of rabies cases in animals and humans in Nepal have been reported from Terai regions compared to hilly and mountain areas of Nepal, which shares an open border with India and have numerous unfenced wildlife areas and densely populated agricultural areas (1, 3, 4). In addition, the highest number of outbreaks were reported in winter (January-February) and summer (June-August), which points toward the possible role of the season on the incidence of rabies as these seasons correspond with the breading season of dogs and other wild canines (7). Similar observation of warm and rainy season having a high incidence of rabies compared to dry season was reported in China (8). Interestingly, the colonies of the Indian Flying Fox (*Pteropus medius*) are common in many parts of Nepal (9), including major cities such as Kathmandu, which can be asymptomatic rabies virus carriers. Although these bats normally carry bat-associated lyssavirus and do generally not carry the classical rabies virus, these bats may be asymptomatic rabies virus carriers and shed the virus in their saliva for months, as is the case with vampire bats (*Desmodus rotundus*) (10–13). These bat colonies may transmit the virus to domestic animals and humans through a cross-species transmission (CST) event as these bat colonies may enter residential areas at night to search for food and may have direct contact with humans and domestic animals, especially community dogs as these community dogs are reported to hunt flying foxes at night and eat them. However, there is a lack of research in these areas of the sylvatic cycle of rabies, and the possible CST from these bat colonies is still unknown. Thus, we must better understand the transmission of rabies between domestic animals and wildlife and apply control measures to prevent possible spillover.

Apart from these ecological and social conditions, the major hindrance to effective rabies control is inadequate funds and associated financial gaps to implement national rabies control programs. Funding from government authorities is fragmented, with human health receiving more attention than the animal health sector, and thereby getting greater funding. The animal health sector is accorded a low priority and thus receives a much smaller budget. For instance, the Ministry of Health (MoH) received around Rs. 103.08 billion, whereas the Ministry of Agriculture and Livestock Development (MoALD) received Rs. 55.97 billion for the fiscal year 2022/23 (14). Out of the total budget of 55.97 billion allocated for MoALD, 15 billion was allocated for procuring chemical fertilizer, so the budget for agricultural purposes would be around 40.97 billion (14), which is 60% less budget than the MoH. This low budget hinders animal disease control programs, including rabies control programs in the animal health sector. In addition to a low budget, a few other issues have hampered the implementation of a national rabies control program. Firstly, funding is only sufficient to respond reactively to outbreaks when in reality, sufficient funds are required to vaccinate widely and prevent the outbreaks from occurring in the first place. Secondly, and more importantly, due to the high level of poverty, many people in rural areas cannot afford the cost of pre-and post-exposure prophylaxis, which is one of the reasons why the uptake of rabies vaccine is very low in rural areas, impeding rabies control in Nepal.

Thirdly, different concerned ministries did not work together to control rabies in Nepal. Rabies control programs have customarily been headed by the Department of Livestock Services (DLS) and animal health professionals, whereas it has been neglected by the human health sectors such as the MoH and the Department of Health Services (DoHS). In addition, wildlife biologists and environmental scientists are not actively involved in rabies control as the current national rabies control programs have not included rabies in the wildlife sectors. All this lack of inter-sectoral collaboration among different concerned stakeholders of rabies control is due to distrust between competing government ministries. To foster intersectoral collaboration among different stakeholders, including different line ministries, Nepal should take a lesson from the successful collaboration models of the OH approach practiced by Rwanda involving community engagement, education, and international collaborations (15), inclusion of rabies control in a national priority and address of problem through multi-sectoral OH approach with a strong political commitment and appropriate budget allocation in Bangladesh (16) and the strengthened OH approach in the Goa state of India (17) that have successfully controlled rabies and adapt those lessons learned to devise and implement preventive and control measures. The local government bodies should be actively engaged in developing effective control measures and improving their diagnostic and reporting capabilities by allocating adequate resources and uninterrupted funding.

Thus, issues identified in the current rabies control programs show that new rabies control programs are needed that take into account the prevailing local ecological and socio-economic conditions to effectively control rabies. However, to do this we need to capture these above-mentioned dimensions that act as predisposing factors and give rise to rabies and use them in rabies control programs. An independent One Health unit could be developed across ministries with a mandate to both do the research to identify the drivers and pathways of transmission of rabies virus and implement those research findings to devise and implement innovative strategies to block rabies virus chain of transmission in animal hosts and subsequently, reduce the disease incidence in humans. Nepal would benefit from the in-depth analysis of the role of ecological and socio-economic drivers on the prevalence and transmission of rabies virus and using those research outputs to inform a national rabies control program and devise a revised evidence-based rabies control program to meet the vision of the zero by thirty rabies strategy. For this, it is necessary to mobilize and align scientific experience, financial resources, and political will and develop a sustainable investment strategy from all concerned stakeholders in Nepal, linked to a regional approach and following the guidelines of international organizations.

## Author contributions

KPA devised the initial idea in consultation with DKY. KPA wrote the initial draft, which was revised by RK, SHC, and DKY. All authors contributed to the article and approved the submitted version.
